# Effect of different isometric trunk extension intensities on the muscle stiffness of the lumbar and lower limbs

**DOI:** 10.3389/fphys.2023.1337170

**Published:** 2024-01-04

**Authors:** Yuting Zhang, Mengtong Chen, Hongxiu Liu, Yanan He, Yuanchao Li, Peifeng Shen, Yiming Chen, Jiapeng Huang, Chunlong Liu

**Affiliations:** Clinical Medical College of Acupuncture, Moxibustion, and Rehabilitation, Guangzhou University of Chinese Medicine, Guangzhou, Guangdong, China

**Keywords:** MyotonPRO, erector spinae, stiffness, isometric prone trunk extension, coordinated contraction

## Abstract

**Purpose:** To investigate the effect of isometric prone trunk extension (IPTE) contraction intensity on the stiffness of erector spinae (ES), semitendinosus (ST), biceps femoris (BF), and gastrocnemius muscles to understand the overall muscle mechanical behavior during IPTE and to explore the mechanisms of oordinated contraction of the body kinetic chain.

**Methods:** Twenty healthy females were recruited, and participants underwent IPTE at three contraction intensities, i.e., 0% maximum voluntary isometric contraction (MVIC), 30% MVIC, and 60% MVIC, and muscle stiffness was measured using MyotonPRO.

**Results:** Muscle stiffness was moderately to strongly positively correlated with contraction intensity (*r* = 0.408–0.655, *p* < 0.001). The percentage increase in stiffness at low intensity was much greater in ES than in lower limb muscles and greater in ST and BF than in gastrocnemius, whereas at moderate intensity, the percentage increase in stiffness decreased in all muscles, and the percentage increase in stiffness in ES was lower than that in ST. There was a moderate to strong positive correlation between ES stiffness variation and ST (*r* = 0.758–0.902, *p* < 0.001), BF (*r* = 0.454–0.515, *p* < 0.05), MG (*r* = 0.643–0.652, *p* < 0.01), LG (*r* = 0.659–0.897, *p* < 0.01).

**Conclusion:** IPTE significantly affected the stiffness of lumbar and lower limb muscles, and low-intensity IPTE activated the ES more efficiently. There were significant coordinated muscle contractions between ES, ST, and LG. This provides preliminary evidence for exploring the overall modulation pattern of the lumbar and lower limb muscles’ kinetic chains. In future studies, we will combine other stiffness assessment methods (such as Magnetic Resonance Elastography, Shear Wave Elastography, or electromyography) to corroborate our findings.

## Introduction

Core strength plays a supportive and protective role in the human body and allows for optimal force production, transmission, and control throughout the body’s kinetic chain during movement (Kibler et al., 2006). Research has shown that there is a kinetic chain between the trunk and the lower extremities (Waiteman et al., 2022) and that the trunk is the basis for the transmission and dissipation of the entire lower extremity kinetic chain during movement. Trunk stabilization is a prerequisite for effective transmission of the spine-pelvis-leg mechanism (Snijders et al., 1993), and the ES is an essential muscle for maintaining upright posture and assisting lumbar spine movement (Sánchez-Zuriaga et al., 2010), and it is the main provider of trunk stability (Guo et al., 2012). The thoracolumbar fascia covers the muscles of the lower trunk (Vleeming et al., 1995), and plays an important role in trunk rotation, lateral flexion, extension, and stabilization of the lower lumbar spine and pelvis. The gluteus maximus (GMax) is a strong hip extensor muscle that is tightly connected to the ES through the thoracolumbar fascia and to the hamstrings through the sacrococcygeal ligament (Vleeming et al., 1995), and the ST in the hamstrings is connected to the gastrocnemius muscle through the fascial bands at the knee joint (Tuncay et al., 2009). Maintaining stability during human movement requires co-activation across muscle groups (Ippersiel et al., 2023). It has been documented that the abdominal and pelvic floor muscles are co-activated during functional activities such as lifting the head and shoulders (Bø and Stien, 1994). During walking or horseback riding (Luzum et al., 2023), co-contraction of the lumbar and lower limb muscles is also required. Muscle co-activation, or co-contraction, is a biomechanical index that quantifies the level of muscle activation around a joint (Rosa et al., 2014), and this coordinated or muscle-activation relationship is defined as coordinated muscle contraction (Sapsford et al., 2001). Altered motor control and coordinated muscular contraction are fundamental to normal body function. Functional activities such as trunk extension require trunk stability (Smith et al., 2021), so the muscle groups that make up the functional unit of trunk extension must contract or co-activate simultaneously, thereby triggering a whole-body muscular response (Kocyigit et al., 2023). However, fewer articles have been published examining the mechanical behavior of trunk and lower limb muscles and the mechanisms of coordinated muscle contraction. Therefore, there is a need for clinical trials on the coordinated contraction of the kinetic chain of the human trunk and lower limb muscles.

Weakened trunk muscle function can lead to overcompensation of other muscles, which can impair athletic performance and even increase the risk of overall musculoskeletal injury (De Blaiser et al., 2018). The third lumbar vertebra (L3), the pivot of lumbar spine movement in all directions, is subject to the greatest stresses, and the muscles attached around it, such as the ES, are among the most vulnerable of the lumbar muscles (Yang et al., 2023). Lumbar muscle conditioning exercises (Clark et al., 2002) can improve the stability of the lumbar structure, effectively enhance the strength and flexibility of the lumbar muscles (San Juan et al., 2005), and enable the lumbar structure to obtain a new balance to compensate for the dysfunction caused by low back pain. Exercises for the lumbar extensors are usually based on isometric exercises to maximize the recruitment of the lumbar muscles (Skibski et al., 2023). An activity level of 60% MVIC is sufficient for basic strength training (Andersson et al., 1998). Muscle activity levels of 20%–30% MVIC have been reported for trunk extensors in daily life (Sawai et al., 2004), and the average intensity of ES contractions is usually moderate or mild (Cuesta-Vargas and Gonzalez-Sanchez, 2013). After pre-experimentation, three contraction intensities of 0%, 30%, and 60% MVIC were selected in this study to explore the optimal intensity for effective ES activation. Trunk flexion produces greater lumbar extension forces than the neutral trunk position, but this is primarily a force produced by the hip extensors with less force being generated by the lumbar muscles (CHOLEWICKI and VANVLIET, 2002; Kocjan and Sarabon, 2014) also found that the lumbar back muscles contributed most to spinal stability in neutral isometric extension exercises compared to flexion tests. In combination with our previous experiment (Zhang et al., 2023), lumbar extension strength in the trunk hyperextension position was not as strong as in the trunk neutral position, and therefore we chose to perform IPTE in the trunk neutral position to assess lumbar extensor strength.

The core is the basis for stabilization and force production in all physical activities, and assessment of core strength allows assessment of muscle functional status (Jubany et al., 2015), which is valuable in the prevention of primary and secondary injuries. Portable ergometers are highly reliable and valid, and they are a suitable method for the rapid assessment of muscle strength in a clinical setting (Stark et al., 2011). Studies have shown that the strength of the tester affects the reliability of hand-held ergometers (Mentiplay et al., 2015), which require the tester to exert a greater force than the muscle being assessed and to apply constant resistance throughout the test (Stark et al., 2011). To eliminate the effect of inter-tester differences in upper limb strength, this study utilized an adjustable fixation device to hold the ergometer in place. Muscle stiffness reflects the resistance to external perturbations, and compared to shear wave elastography MyotonPRO can provide stiffness parameters of individual muscles during isometric contractions of varying intensities with excellent reliability (Lam et al., 2015). Rapid quantification of overall spatially distributed muscle stiffness at rest and at different contraction intensities can help us better understand muscle recruitment strategies and provide new insights into the mechanisms of coordinated contraction of the kinetic chain between the lumbar and lower limb muscles.

The purpose of this study was to quantify the changes in stiffness of the right ES, ST, BF, medial gastrocnemius head (MG), and lateral gastrocnemius head (LG) during non-fatiguing IPTE of varying intensities in healthy participants by using MyotonPRO.

## Materials and methods

### Sample size calculation

To set the required sample size for the study, five participants were first screened according to the inclusion and exclusion criteria for the pre-experiment, which showed that the stiffness of erector spinae at 0% of the contraction intensity of trunk extension was 270.53 ± 2.79 N/m, that of erector spinae at 30% of the contraction intensity was 348.93 ± 11.69 N/m and that of erector spinae at 60% of the contraction intensity was 380.33 ± 15.99 N/m. Requiring bilateral α = 0.05 and power = 0.95, the software G*Power3.1.9.7 (Heinrich-Heine-Universität, Düsseldorf, Germany) calculated the effect size of ES to be 2.89, with a minimum sample size of 9. Adequate consideration was given to the existence of the possibility of participant refusal and loss, and a total of 20 participants were recruited for this study.

### Ethical approval

This study received approval from the ethics committee of the Guangdong Provincial Hospital of Chinese Medicine (YF 2021-223-01). This study followed the principles of the Declaration of Helsinki. All participants were fully informed about the safety of the ergometer and MyotonPRO, their basic rights, the purpose of the trial, and the procedure before the start of the trial, and they signed an informed consent form in writing.

### Participants

The researchers recruited 20 healthy females (mean age: 19.95 ± 0.95 years; mean height: 1.60 ± 0.05 m; mean weight: 50.75 ± 6.14 kg; BMI: 19.69 ± 1.83 kg/m^2^; MVIC: 134.53 ± 5.85N) from Guangzhou University of Chinese Medicine in June 2023. Inclusion criteria were 1) right hand and foot were the dominant sides; 2) 18.5 kg/m^2^ < BMI <24 kg/m^2^; 3) being in good health without neuromuscular disease or joint disease and history of spinal or pelvic surgery; and 4) not having pain or trauma in the shoulder and neck, low back, lower limbs, or feet of those who had not had pain or trauma that interfered with normal life and work for at least the past 6 months. Exclusion criteria were: 1) those with broken skin or bleeding tendency in the lumbar and lower limbs; 2) cognitive impairment; 3) pregnancy, breastfeeding, menstruation, and women with prolonged dysmenorrhea; and 4) scoliosis. Participants were asked to avoid strenuous activity for 48 h before the start of the trial.

### Equipment and parameter settings

This study was conducted using MyotonPRO (produced by MyotonPRO AS in Estonia). The operator places the probe vertically against the skin surface of the marking point, presses the body gently until the green light at the end of the probe illuminates, and stabilizes the instrument until the probe automatically impacts the measurement and the muscle dynamic hardness, i.e., S-Dynamic Stiffness (N/m), is displayed on the screen of the device. The ergometer is a HOGGAN Scientific microFET2 (Hoggan Scientific, Salt Lake City, UT, United States) used to measure ES contraction strength, which is converted from a pressure signal into pounds by a pressure transducer and displayed on the screen.

### Experimental protocol

**FIGURE 1:**
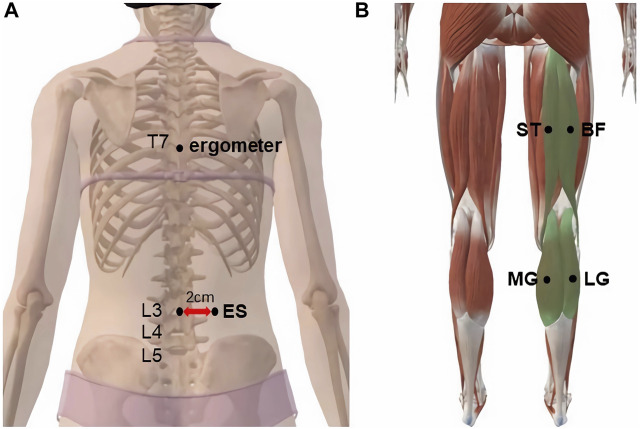
MyotonPRO probe monitoring point: **(A)** erector spine; **(B)** semitendinosus, biceps femoris, medial gastrocnemius, and lateral gastrocnemius.

In this test, MyotonPRO was used to measure the stiffness of the right ES, ST, BF, MG, and LG. The operator marked the muscles to be measured with an oil pen, palpated the spinous process of the fourth lumbar vertebra (L4) according to the body markers, palpated L3 upward, and marked the ES measurement point 2.5 cm to the right lateral paracentesis of L3. The measurement point of the ST was the midpoint of the line connecting the sciatic tubercle to the medial tibial condyle; that of the BF was the midpoint of the line connecting the sciatic tubercle to the lateral tibial condyle; the MG was located at 30% of the line connecting the medial popliteal stripe with the lateral ankle; and the LG was located at 30% of the line connecting the lateral popliteal stripe with the medial ankle (Figure 1).

**FIGURE 2:**
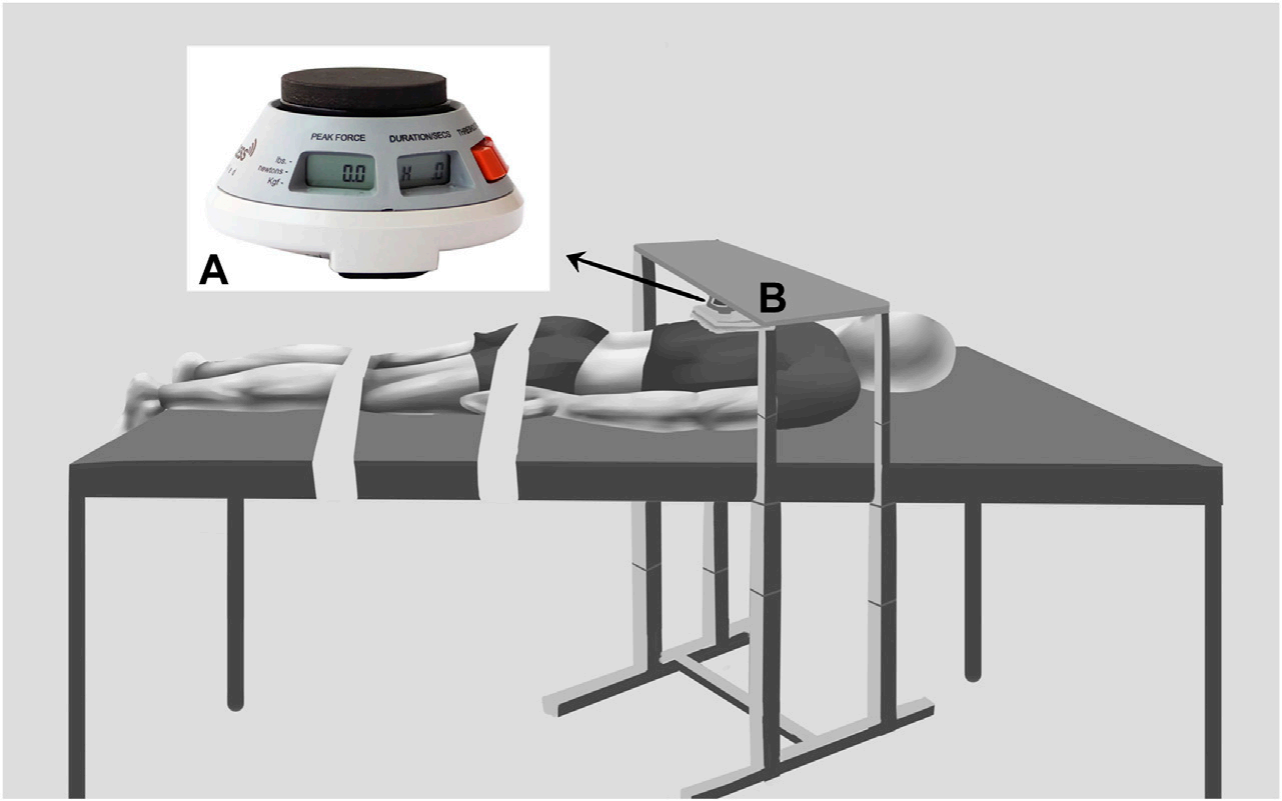
The posture and equipment used for measurement: **(A)** ergometer, **(B)** Adjustable fixtures.

The test steps were as follows: 1) During the measurement period, the participant lay prone on a full-length padded manipulative bed with the arms parallel to the body axis, the lower limbs straight and relaxed, and the ankles extended outside the bed in a naturally drooping position. Two immobilization belts secured the participant to the manipulative bed. The band that immobilized the pelvis was located at the level of the greater trochanter of the hip, and the band that immobilized the lower extremities was placed at the level of the popliteal fossa. The adjustable fixtures secured the ergometer (Figure 2) at the seventh thoracic spine spinous process (T7) to assess the strength of the ES during IPTE. A towel was used between the ergometer and the T7 spinous process to minimize participant discomfort. After 5 min of rest in the prone position, the operator measured the ES, ST, BF, MG, and LG stiffness on the dominant side of the participant using a MyotonPRO; 2) the participant was asked to perform three voluntary movements of maximal isometric trunk extension, each of which was 5 s with a 45 s rest period, and the three times were recorded to take the average ergometer value, which was 100% MVIC. The resistance required for contraction was calculated as follows: 30% MVIC = 0.3 MVIC and 60% MVIC = 0.6 MVIC; 3) after 10 min of rest, the participant was asked to maintain isometric trunk extension at 30% and 60% MVIC, respectively, and the operator took 3 repetitive measurements and averaged the values of the ES, ST, BF, MG, and LG using the MyotonPRO with the order of the muscle measurements randomized. During each contraction, the MicroFET2’s display shows the resistance value and the operator verbally instructs the participant to remain at the target resistance value. The target value threshold ranges from +/- 2.5% to account for variance observed in contraction control. The operator measures two to three muscle positions at a time for approximately 20–30 s. As soon as the MicroFET2 real-time muscle strength data showed a wide range of fluctuations in the participant’s muscle strength (more than +/- 2.5%), we stopped collecting data and asked the participant to rest appropriately between measurements to recover from fatigue and to monitor muscle stiffness using MyotonPRO until it returned to its initial state.

### Statistical analysis

**FIGURE 3:**
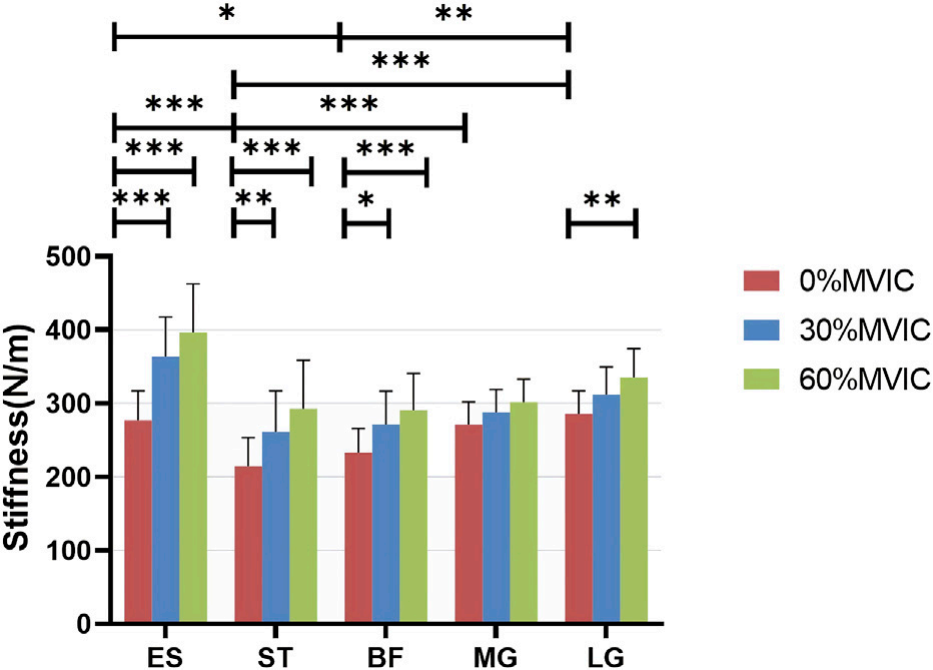
Stiffness variation of each muscle under different contraction intensities. ***, significant intergroup difference (*p* < 0.001); **, significant intergroup difference (*p* < 0.01); *, significant intergroup difference (*p* < 0.05); NS, non-significant intergroup difference (*p* > 0.05).

SPSS 25.0 (version 25.0, Chicago, IL, United States) was used for statistical analysis. All data collected were tested for normality using the Shapiro-Wilk test, and all data conformed to a normal distribution and are expressed as mean ± standard deviation (SD). One-way analysis of variance (ANOVA) was performed with 0% MVIC, 30% MVIC, and 60% MVIC as independent variables and muscle stiffness (same measurement position) as the dependent variable. When ANOVA results were significant, Tukey’s multiple comparisons were performed to determine whether there was a difference in the stiffness of the same muscle at different contraction intensities (Figure 3). The statistical significance level was set at *p* = 0.05.

Pearson correlation analysis was used to verify the correlation between muscle hardness and isometric contraction strength, as well as the correlation between the value of the percentage change in muscle hardness of the lower limbs and ES. The correlation strength of the correlation coefficient r-value was set as |r| < 0.3 for weak correlation, 0.3 ≤ |r| ≤ 0.6 for moderate correlation, and |r| > 0.6 for strong correlation. Considering the individual differences in subjects and the stiffness properties of the muscles themselves, localized muscle stiffness percentage changes were used to more accurately characterize the differences in stiffness during contraction and force production. Percentage change in stiffness (%) = (measured stiffness—initial stiffness) ÷ initial stiffness × 100%. The percent change in muscle hardness at each measurement location was compared to the percent change in ES hardness under the same test conditions. The statistical significance level was set at *p* = 0.05.

## Results

### Comparison of the stiffness of lumbar and lower limb muscles at different isometric trunk extension contraction intensities

Table 1 lists the Sum of Squares, Mean Square, F-values, and *p*-values for ES, ST, BF, MG, and LG stiffnesses at different isometric trunk extension contraction intensities (0%, 30%, and 60% MVIC). The ANOVA test showed extremely statistically significant differences in ES, ST, BF, and LG stiffness at different trunk extension intensities (*p* < 0.001), as well as statistically significant differences in MG stiffness as well (*p* = 0.012). Figure 3 shows the relationship between muscle stiffness and the intensity of contraction for different isometric trunk extensions. Post-hoc Türkiye test showed that different muscles had different stiffness in a relaxed state: ES differed from ST (*p* = 0.002) and BF (*p* = 0.0182), ST differed significantly from MG (*p* = 0.0009) and LG (*p* < 0.0001), and BF differed from LG (*p* = 0.0025). The same muscle also differed in stiffness at different contraction intensities, with ES, ST, and BF stiffness all differing significantly at both 0% MVIC and 30% MVIC, and 0% MVIC and 60% MVIC (*p* < 0.0001, *p* < 0.0001, *p* = 0.0001, *p* = 0.0038, *p* < 0.0001, *p* = 0.0196, and *p* = 0.002), and LG stiffness differing between 0% MVIC and 60% MVIC (*p* = 0.0016), and there was no significant difference in muscle hardness at the remaining contraction intensities (*p* > 0.05). The same tester performed all stiffness measurements, and the reliability analysis resulted in excellent intragroup reliability (intra-class correlation = 0.985–0.993 for 5 muscle sites).

**TABLE 1:** One-way ANOVA results of muscle stiffness at different isometric trunk extension contraction intensities (mean ± SD, *n* = 20).

Independent variables	Position	Stiffness (N/m)	Sum of squares	Mean square	F	*p*-values
% MVIC (0%, 30, 60%)	ES	345.61 ± 73.86	152,540.293	76,270.146	25.673	0.000
	ST	255.97 ± 62.81	61,778.115	30,889.057	10.298	0.000
	BF	265.01 ± 48.98	34,937.955	17,468.978	9.342	0.000
	MG	286.67 ± 33.12	9,234.233	4,617.117	4.743	0.012
	LG	310.76 ± 41.12	24,798.337	12,399.169	9.429	0.000

SD, standard deviation; ES, erector spinae; ST, semitendinosus; BF, biceps femoris; MG, medial gastrocnemius; LG, lateral gastrocnemius.

### Characteristics of muscle stiffness and percentage change in stiffness at different contraction intensities

A moderate to strong positive correlation between muscle stiffness and isometric contraction intensity (*r* = 0.408–0.655, *p* < 0.001) is shown in Table 2. The stiffness of ES, ST, BF, MG, and LG contractions of the participants increased with increasing intensity of trunk extension (Table 3; Figure 3).

**TABLE 2:** The correlation between muscle stiffness and isometric contraction intensity.

Measurement position	*r*-values	*p*-values
ES	0.655	0.000
ST	0.503	0.000
BF	0.465	0.000
MG	0.408	0.001
LG	0.487	0.000

SD, standard deviation; ES, erector spinae; ST, semitendinosus; BF, biceps femoris; MG, medial gastrocnemius; LG, lateral gastrocnemius.

**TABLE 3:** Changes in muscle stiffness and percentage changes in stiffness of lumbar and lower limb muscles under different isometric contraction intensities (mean ± SD, %).

Measurement position	Contraction intensity	Stiffness (N/m)	Percentage change (%)		
			0–30% MVIC	30–60% MVIC	0–60% MVIC
ES	0% MVIC	276.88 ± 40.01	32.13 ± 15.97	8.84 ± 3.74	44.04 ± 19.58
	30% MVIC	363.52 ± 54.18			
	60% MVIC	396.43 ± 66.15			
ST	0% MVIC	214.55 ± 38.56	21.21 ± 11.77	12.15 ± 5.31	35.99 ± 15.28
	30% MVIC	260.63 ± 56.19			
	60% MVIC	292.73 ± 65.99			
BF	0% MVIC	232.80 ± 33.00	16.42 ± 7.80	7.20 ± 3.52	24.86 ± 10.38
	30% MVIC	271.33 ± 44.99			
	60% MVIC	290.88 ± 49.97			
MG	0% MVIC	270.85 ± 31.05	6.45 ± 2.99	4.60 ± 1.98	11.36 ± 3.97
	30% MVIC	288.00 ± 30.77			
	60% MVIC	301.15 ± 31.77			
LG	0% MVIC	285.25 ± 31.63	9.38 ± 5.01	7.43 ± 2.89	17.50 ± 5.9
	30% MVIC	312.02 ± 37.40			
	60% MVIC	335.00 ± 39.32			

SD, standard deviation; ES, erector spinae; ST, semitendinosus; BF, biceps femoris; MG, medial gastrocnemius; LG, lateral gastrocnemius; MVIC, maximum voluntary isometric contraction.

**FIGURE 4:**
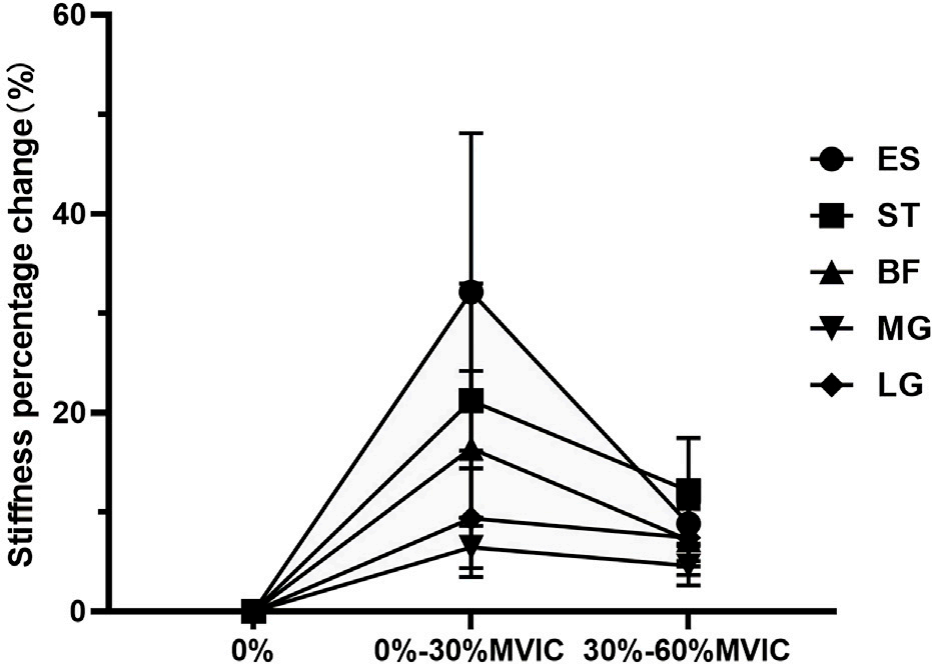
The change in stiffness percentage changes value of each muscle under different contraction intensities.

The characteristics of muscle stiffness percentage change are shown in Table 3; Figure 4. When the contraction intensity was 0%–30% MVIC, the values of percentage change of muscle stiffness increased in both lumbar and lower limbs, and the degree of increase of percentage change values was ES > ST > BF > LG > MG; when the contraction intensity was 30%–60% MVIC, the percentage growth of stiffness decreased in all muscles, and the value of percentage change of stiffness growth in ES decreased sharply, and the degree of increase of percentage change values was ST > ES > LG > BF > MG.

### Correlation between ES and lower limb muscle stiffness at different muscle contraction intensities

The percentage of muscle stiffness at each measurement position was compared to the percentage of stiffness at the ES under the same test conditions (Table 4). The results showed that ST (*r* = 0.758–0.902, *p* < 0.001), LG (*r* = 0.659–0.897, *p* < 0.01), and MG (*r* = 0.643–0.652, *p* < 0.01) showed strong correlations with the values of percent change in stiffness of the ES at different contraction intensities. bf (*r* = 0.454–0.515, *p* < 0.05) was moderately correlated with the value of the percent change in stiffness of ES.

**TABLE 4:** The correlation between the stiffness percentage changes of each muscle (%).

Measurement position	0–30% MVIC		0–60% MVIC	
	*r*-values	*p*-values	*r*-values	*p*-values
ES	1	—	1	—
ST	0.902	0.000***	0.758	0.000***
BF	0.515	0.020*	0.454	0.044*
MG	0.652	0.002**	0.643	0.002**
LG	0.897	0.000***	0.659	0.002**

ES, erector spinae; ST, semitendinosus; BF, biceps femoris; MG, medial gastrocnemius; LG, lateral gastrocnemius; MVIC, maximum voluntary isometric contraction. ***, significant intergroup difference (*p* < 0.001); **, significant intergroup difference (*p* < 0.01); *, significant intergroup difference (*p* < 0.05).

## Discussion

To the best of our knowledge, this is the first study to observe the mechanical behavior and kinetic chain coordinated contraction mechanisms of the lumbar and lower limb muscles during IPTE of different intensities and to correlate the percentage change in muscle stiffness of the lumbar and lower limb muscles. This study found the following characteristics of muscle stiffness and percent stiffness change: 1) muscle stiffness was moderately to strongly positively correlated with contraction intensity; 2) there was an inverted U-shaped relationship percentage change in muscle stiffness growth and contraction intensity, the percent increase in stiffness of ES was much greater than that of the lower limb muscles during low-intensity contraction, the percent increase in stiffness of hamstring muscle was greater than that of gastrocnemius muscle, and the percent increase in stiffness of ES was less than that of ST during medium-intensity contraction; 3) there was a moderate to strong correlation between the value of percent change in stiffness of lower limb muscles and ES to strong correlation; 4) there were significant coordinated muscle contractions between ES, ST, and LG.

Skeletal muscle is usually cross-linked with myosin (contractile properties) and actin and connective tissue (passive elastic) components to produce force. The biomechanical properties of muscle can reflect the physiological changes of muscle during relaxation and contraction (Wang et al., 2017). This study used MyotonPRO quantitatively produced in stiffness change of muscle contraction and local muscle stiffness of noninvasive quantitative measurements to estimate individual muscle’s contribution to the overall joint hardness (Nordez et al., 2009). The results showed that there was a moderate to strong positive correlation between skeletal muscle stiffness and non-fatigue contraction strength (0–60% MVIC), which was consistent with the results of many previous studies (Bensamoun et al., 2008; Nordez et al., 2009; NORDEZ and Hug, 2010). Therefore, muscle stiffness can be used to estimate the relative change in muscle strength and degree of contraction.

Core muscles provide proximal stability for the spine and are the stable basis for upper and lower limb movements (Guo et al., 2012). Studying the effect of IPTE on muscle stiffness may clarify the regulation strategies of different muscles. This study found that different muscles have different stiffness in the relaxed state, which may be related to the direction of muscle fibers (Bensamoun et al., 2006). By analyzing the percentage changes in the stiffness of each muscle under different contraction intensities, we found that the stiffness of different muscles increased unevenly with the increase in contraction intensity. During low-intensity contraction, the percent increase in stiffness of ES, ST, and BF was much higher than that of MG and LG, which indicates that ES is the main generating site of the lumbar extensor muscle, ST is the main generating muscle of the hip extensor muscle, and it is also the lower limb muscle most affected by changes in lumbar tension. This finding also supports the hypothesis of Arokoski et al. (1999). However, during moderate-intensity contraction, the percentage increase in ST stiffness was greater than that of ES, indicating that the synergistic relationship between the lumbar extensor and hip extensor also changed with the increase in load. Clark et al. (2002) used surface electromyography to observe the effects of multiple sets of dynamic PTE at 40%, 50%, and 70% MVIC exercise intensities on the electromyography activities of the para-spinal area, GMax, and BF. The results showed that when the exercise intensity was greater than 40% MVIC, the contribution of the hip extensor muscle to the force was greater than that of the lumbar extensor muscle, and the hip extensor muscle was more adaptable to high loads than the lumbar muscle tissue. Saunders et al. (2020) found that continuous IPTE produced less head movement than multi-group dynamic PTE and that head movement was linearly related to ES contraction intensity, with the least head movement and the most stable ES contraction at 30% MVIC. The above findings are consistent with the results of the present study, in which the output strategies of the lumbar and hip extensors were redistributed at 60% MVIC and the ST contributed more to trunk extension force generation than the ES. Therefore, to activate the lumbar extensor muscle more effectively and minimize the synergistic effect of the lower limb muscles, we should choose low-intensity IPTE to train ES, that is, 30% MVIC.

Our study found an inverted “U”-shaped relationship between percentage change in muscle stiffness growth and contraction intensity. It has been shown (Taylor et al., 2003) that at low loads, motor units are largely inactive, so any change in the recruitment of motor units causes high fluctuations. The relative increments in the recruitment of each motor unit decreased with increasing contraction intensity, and the fluctuations reached a plateau with further increases in the force level. Compared with low-intensity contraction, the percentage increase in ES stiffness decreases sharply during medium-intensity contraction. This is due to the relatively high proportion of slow fibers, i.e., type I muscle fibers, in the lumbar muscles (Mannion et al., 1997), which mainly provide spinal stability (Panjabi et al., 1989). Synergistic muscles such as hip extensors are activated to prevent overloading of the lumbar spine region (Clark et al., 2002). With the increase in contraction intensity, the percentage increase in stiffness in ST was always greater than that in BF, mainly because ST is a fusiform muscle with longer muscle fiber length (Eleftherios, 2018), and fusiform muscle is considered to be beneficial for muscle contraction (WOODLEY and SUSAN, 2005). The fascicle length/muscle length ratio of ST is almost doubly normalized (relative to sarcomere length), and ST has greater offset capacity and compliance compared to BF (Kellis et al., 2012). Moreover, compared with BF, ST has a relatively high proportion of fast fibers, namely, type II fibers (Smith et al., 2011), so ST can achieve faster contraction. The percentage increase in stiffness of LG was also consistently greater than that of MG, and may also be related to the type of fiber that makes up the muscle. MG usually consists of a higher proportion of type I fibers (Johnson et al., 1973), and LG consists of more type II fibers, so LG can achieve faster contraction.

In the kinetic chain theory, when we move a certain part of the body, the parts adjacent to it or far away from it will be affected to some extent, and the cooperation of many joint and muscle movements produces an overall complex movement. If muscle tension can change depending on the characteristics of a particular movement, then this may be a manifestation of the synergistic contraction of adjacent or distant muscle groups required to accomplish that movement (Krause et al., 2016). Wilke J et al. (2016) have shown that this applies to muscles in the longitudinal series of superficial back line. The superficial back line increases trunk stability by integrating tension in the body’s core and the limbs, promoting coordinated muscle contraction (Krause et al., 2016). Studies have shown that local tissue stretching generates substantial force transmission between adjacent parts that are parallel (such as the gastrocnemius and soleus) or in series (such as the hamstring and gastrocnemius) (Vleeming A et al., 1995; Thorstensson and Carlson, 1987) found in an *in vivo* study that the deep fascia of the gastrocnemius muscle could be displaced during pelvic movement. The study by (Vleeming et al., 1995) pointed out that GMax is anatomically connected to the ES via TLF, forming a kinetic chain that results in co-contraction of the lumbar and pelvic muscles during trunk movements. Stecco et al. (2013) extended the load transfer from the pelvis to the knee because the fascia of GMax inserts into the iliotibial band and lateral muscle septum. In this way, there may be kinetic chains between the lumbar and pelvic regions up to the knee joints. Wilke et al. (2020) found that moving the ankle during knee extension caused caudal displacement of the hamstring muscle. Snoeck et al. (2014) found that ST and gracilis (G) were crucial in forming the body kinetic chain and causing the displacement of the calf muscles. In addition, anatomical studies found that there was a fascial connection between the gastrocnemius muscle and the ST (Wilke et al., 2016). These non-local changes may be the result of coordinated muscle contractions along the kinetic chain, and the extent of long-distance motion effects caused by coordinated muscle contractions may depend on the stiffness of the continuity of the affected tissue, studying the correlation of stiffness changes can be used as an alternative test of force transmission (Mohr et al., 2023). It can be seen from the above situation that there is tissue continuity in the major muscle groups activated during IPTE training with different contraction strengths, and there is a coordinated contraction of the kinetic chain between ES, hamstring, and gastrocnemius, which is mainly manifested as ES, ST, and LG, which is consistent with the results of our previous experiment (Zhang et al., 2023).

This study explored the changes and correlations of lumbar and lower limb muscle stiffness under different IPTE contraction intensities, which provided a reference for us to understand how to train lumbar muscles more effectively and prevent injury, elucidated the coordinated contraction effect of the human kinetic chain, and clarified the adjustment strategy and mechanical behavior of muscles during trunk extension.

### Limitations

This study has some limitations. Firstly, because the depth of GMax exceeded the measurement threshold of MyotonPRO, we were unable to measure the hardness of GMax in this study, and we were not able to observe *in vivo* the role of GMax in coordinated contraction in the waist and lower limbs. Secondly, although MyotonPRO has excellent reliability in measuring muscle stiffness, its validity has not been supported by evidence. Thirdly, the current experimental results only apply to the muscle stiffness properties of young females and cannot determine the mechanical properties of muscle contraction in other populations. Future experiments will be repeated with different age and gender groups. Finally, only healthy people were included in this study, and related studies on people with low back pain will be gradually carried out in the future.

## Conclusion

In this study, we found that ES and lower limb muscles exhibited different muscle mechanical behaviors at different contraction intensities of IPTE. Muscle stiffness showed a moderate to strong positive correlation with contraction intensity, with 30% MVIC being the optimal intensity for effective ES activation. ES and lower limb muscle have a moderate to strong correlation between the stiffness percentage change, and there were significant coordinated muscle contractions between ES, ST, and LG. In this study, we provided preliminary evidence that coordinated contraction of the lumbar and lower limb muscles occurs during isometric prone trunk extensions, which provides a further reference for us to understand the overall regulation mode of the muscle kinetic chain. In future studies, we will combine the use of other stiffness assessment methods (e.g., Magnetic Resonance Elastography, Shear Wave Elastography, or electromyography) to corroborate our findings.

## Data Availability

The raw data supporting the conclusion of this article will be made available by the authors, without undue reservation.

## Abbreviations

ANOVA, analysis of variance; BF, biceps femoris; ES, erector spinae; Gmax, gluteus maximus; IPTE, isometric prone trunk extension; L3, the spinous process of the third lumbar vertebra; L4, the spinous process of the fourth lumbar vertebra; LG, the lateral head of the gastrocnemius; MG, the medial head of the gastrocnemius; MVIC, maximum voluntary isometric contraction; SD, standard deviation; ST, semitendinosus; T7, the spinous process of the seventh thoracic vertebra.
